# Transcatheter embolization for idiopathic peripheral pulmonary arterial aneurysm: A case report

**DOI:** 10.1002/ccr3.2154

**Published:** 2019-04-15

**Authors:** Tomoki Tamura, Takayuki Yabuki, Tomoka Kawajiri, Tatsuya Nishi, Kenichiro Kudo, Shoichi Kuyama

**Affiliations:** ^1^ Department of Respiratory Medicine Iwakuni Clinical Center Iwakuni Japan; ^2^ Department of Radiology Iwakuni Clinical Center Iwakuni Japan

**Keywords:** AVP 4, embolization, hydrogel‐coated metallic coil, peripheral pulmonary arterial aneurysm

## Abstract

The natural history of idiopathic peripheral pulmonary arterial aneurysms (PAAs) is unclear; however, they can cause sudden death by rupture. Our case illustrates the utility and low invasiveness of transcatheter embolization using an AMPLATZER™ Vascular Plug 4 and hydrogel‐coated metallic coils in patients with idiopathic peripheral PAAs.

## 1 INTRODUCTION

Pulmonary arterial aneurysms (PAAs) are very rare, the reported incidence being 0.007%.^1^ PAAs are classified according to causes as congenital, acquired, and idiopathic.^2^ Idiopathic PAAs are rare; however, several cases have been reported.^3^, ^4^, ^5^, ^6^, ^7^, ^8^, ^9^ Greene and Baldwin have proposed four pathological criteria for diagnosing an idiopathic PAA: simple dilatation of the pulmonary trunk with or without involvement of the rest of the arterial tree, the absence of intracardiac or extracardiac shunts, the absence of chronic cardiac or pulmonary disease, and the absence of arterial disease such as syphilis or more than minimal atheromatosis or arteriosclerosis of the pulmonary vascular tree.^10^ We here report a patient who underwent transcatheter pulmonary artery embolization for an idiopathic peripheral PAA and had a good clinical outcome.

## 2 CASE REPORT

**Figure 1:**
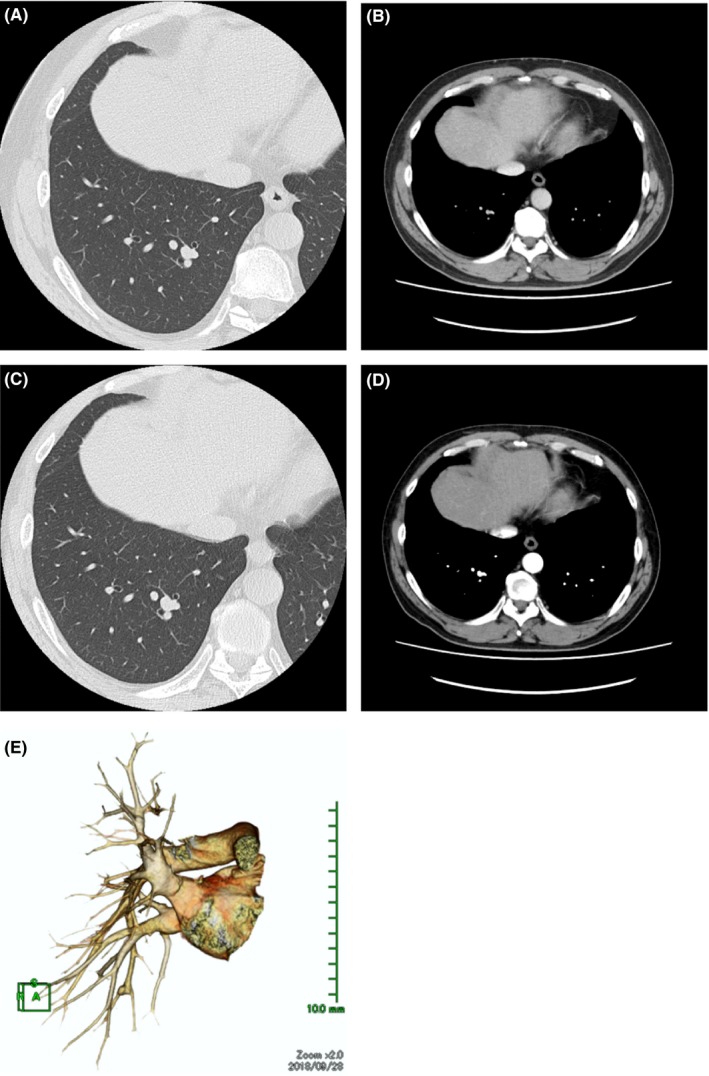
A, High‐resolution computed tomography (CT) scan image showing an 8.6 mm diameter aneurysm in the periphery of right pulmonary artery 10. B, Contrast‐enhanced CT scan images showing an 8.6 mm diameter aneurysm in the periphery of right pulmonary artery 10. C, The aneurysm's diameter has increased to 9.9 mm one year later. D, The aneurysm's diameter has increased to 9.9 mm one year later. E, Three‐dimensional reconstruction of contrast‐enhanced CT scan showing the aneurysm at the branch of A10b and A10c

A 53‐year‐old man was referred to our institution after a peripheral PAA had been discovered by contrast‐enhanced computed tomography (CT) that demonstrated an 8.6 mm diameter aneurysm in the periphery of the right pulmonary artery A10 (Figure 1A,B). He chose follow‐up observation. A CT one year later showed the diameter of the PAA had increased to 9.9 mm (Figure 1C‐E). No abnormality was found on cardiac ultrasound examination, and his tricuspid valve pressure disparity was normal (14 mm Hg). He had the comorbidity of diabetes, which was well controlled. He had a smoking history (Brinkman index 3450); however, his respiratory function was normal. He had no history of infectious diseases such as syphilis or tuberculosis and no history of Behcet disease or Marfan syndrome.

### 2.1 Transcatheter embolization

**Figure 2:**
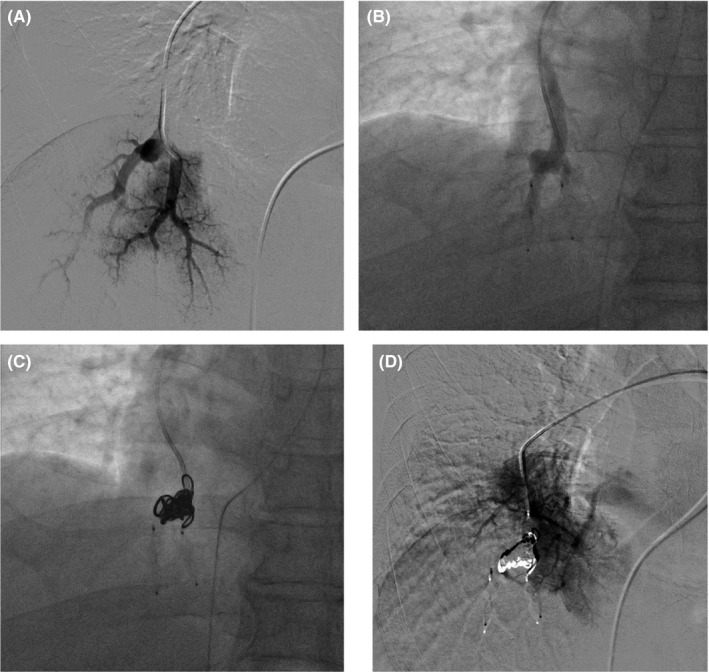
A, Right A10b+c angiogram showing the aneurysm at the branch of A10b and A10c. B, Distal embolization was performed using an AMPLATZER™ Vascular Plug 4. C, The aneurysm was embolized using metallic coils. D, Right A10b+c angiogram showing complete occlusion with no aneurysm apparent

Transcatheter pulmonary artery embolization was performed to prevent rupture of the peripheral PAA. After placement of a 4 Fr introducer sheath in the right femoral vein under local anesthesia, a right pulmonary artery angiogram confirmed the PAA at the branches of A10b and A10c (Figure 2A). To embolize the draining artery, an AMPLATZER™ Vascular Plug 4 (AVP 4; St. Jude Medical) was used. A10b, one of the draining arteries, was plugged with a 6‐mm AVP 4, and A10c, the other draining artery, was plugged with a 7‐mm AVP 4 (Figure 2B). The PAA was embolized with four hydrogel‐coated metallic coils, AZUR^®^ CX35 (Azur peripheral hydrocoil; Terumo Medical Corporation) (Figure 2C). A10b+c, the feeding artery, was plugged with an 8‐mm AVP 4. Occlusion of the PAA was confirmed by repeat angiography after embolization (Figure 2D).

## 3 DISCUSSION

Pulmonary arterial aneurysms are very rare. Deterling and Clagett reported eight patients with PAAs in 109 571 consecutive postmortem examinations.^1^ Depending on the site of onset, PAAs are classified as central and peripheral. The term central PAAs denotes PAAs generated in the left and right main pulmonary arteries from the main pulmonary artery, whereas peripheral PAA denotes PAAs generated from a lobe branch in the periphery. Central PAAs are often secondary to pulmonary hypertension and vasculitis, whereas peripheral PAAs are often secondary to inflammation and trauma associated with arteriosclerosis.^11^ In the present patient, we considered the PAA to be idiopathic because he had none of these underlying diseases. Although secondary PAAs reportedly have a high incidence of secondary hemoptysis resulting from rupture,^6^, ^12^, ^13^ there are few reports of rupture of idiopathic peripheral PAAs. However, in this case the PAA increased. According to Inaba et al, hemoptysis occurs in only one of 12 patients.^6^ In the past, it was recommended that pulmonary aneurysms should be treated as soon as the diagnosis had been made^11^, ^14^; however, the current recommendation is to resect only very invasive idiopathic peripheral PAAs, especially those considered at relatively high risk of rupture. In such patients, intravascular treatment is less invasive and therefore preferable to surgery. Embolization methods include embolization of the aneurysm, embolization of the feeding artery, embolization of both the feeding and draining arteries, occluding the aneurysm, and embolization of both the feeding and draining arteries. The procedure can be shortened by using an AVP to embolize both the feeding and draining arteries.^13^ Though it remains unclear which pulmonary aneurysms should be treated, transcatheter embolization is a minimally invasive means of treating for idiopathic peripheral PAAs. Accumulation of more cases is needed.

## 4 CONCLUSION

Transcatheter embolization using AVP 4 could become the standard means of treating idiopathic peripheral PAAs.

## CONFLICT OF INTEREST

The authors have no conflict of interests to disclose.

## AUTHOR CONTRIBUTIONS

TT: conceived and designed the study, acquired the data, analyzed and interpreted the data, wrote the manuscript, and approved the final manuscript. TY, TK, TN, KK, SK: analyzed and interpreted the data, drafted the manuscript, and approved the final manuscript.

## INFORMED CONSENT

Appropriate written informed consent was obtained for publication of this case report and accompanying images.
